# Neoadjuvant therapy for thymic neoplasms reduces tumor volume per 3D-reconstructed images but does not improve the complete resection rate

**DOI:** 10.1371/journal.pone.0214291

**Published:** 2019-03-26

**Authors:** Jee Won Suh, Seong Yong Park, Chang Young Lee, Seung Hwan Song, Dae Joon Kim, Hyo Chae Paik, Kyoung Young Chung, Min Hee Hong, Hye Ryun Kim, Byoung Chul Cho, Jin Gu Lee

**Affiliations:** 1 Department of Thoracic and Cardiovascular Surgery, Severance Hospital, Yonsei University College of Medicine, Seoul, Republic of Korea; 2 Division of Medical Oncology, Department of Internal Medicine, Yonsei Cancer Center, Yonsei University College of Medicine, Seoul, Republic of Korea; Baylor College of Medicine, UNITED STATES

## Abstract

**Objectives:**

Complete resection of thymic neoplasms is important for achieving a favorable prognosis; however, the efficacy of neoadjuvant therapy remains controversial. We investigated the effect of induction therapy on complete resection and survival using 3-dimensionally reconstructed images to measure tumor volume.

**Methods:**

Eighty-nine patients who underwent surgical resection for Masaoka-Koga stage III–IV thymic neoplasms between January 2000 and December 2013 were enrolled, including 71 and 18 in the primary surgery and neoadjuvant therapy groups, respectively. Baseline characteristics, postoperative outcomes, and survival rates were analyzed. Moreover, baseline and post-neoadjuvant therapy tumor volumes were compared among patients in the neoadjuvant group.

**Results:**

Adjacent mediastinal structure invasion was significantly rarer in the primary surgery group than in the neoadjuvant group (1.27±1.09 vs. 2.61±1.42, p<0.001). On subgroup analysis of patients who underwent neoadjuvant therapy, tumor volumes decreased significantly from 206.08±132.32 cm^3^ to 81.25±71.24 cm^3^ post-therapy (p = 0.001). Interestingly, only the pre-neoadjuvant tumor volume was significantly associated with complete resection, while the post-neoadjuvant volume was not (p = 0.012 and p = 0.458, respectively). Moreover, despite significantly reduced tumor volumes, patients in the neoadjuvant therapy group did not exhibit significantly different R0 resection rates (odds ratio 1.490, p = 0.581) or overall survival (p = 0.285) compared to those in the primary surgery group.

**Conclusions:**

Neoadjuvant therapy does not significantly influence the R0 resection rate or overall survival relative to primary surgery. Nevertheless, it may by useful for patients planning surgical resection because it significantly reduces the presurgical tumor volume and extent of invasion.

## Introduction

Thymic neoplasms are rare tumors with an annual incidence of 1–5 per million people [1]. According to the International Thymic Malignancy Interest Group, approximately 30% of thymic neoplasms are of Masaoka-Koga stage III–IVa [2]. However, the optimal treatment for advanced thymic tumors remains controversial [3]; in patients with locally advanced disease, achieving complete resection is a critical determinant of recurrence and overall survival [4]. That patients with locally advanced thymic tumors have poorer outcomes than those with early-stage disease is evidence for the clinical necessity of multimodal approaches [5]. Cisplatin-based induction chemotherapy is generally well tolerated, and patients with advanced thymic tumors have exhibited favorable responses to it in several prospective clinical trials [4, 6]. A previous meta-analysis revealed that thymomas exhibited marked chemosensitivity with clinical response rates of 49–70%; furthermore, complete resection rates ranged from 67% to 79% [4].

Previous studies on the effects of neoadjuvant therapy relied on the use of 2-dimensional computed tomography (CT) images to determine the maximum tumor size [7]. However, we hypothesized that tumor volume, rather than tumor diameter, was more closely associated with complete resection. Therefore, we performed this study to evaluate the effect of neoadjuvant therapy on tumor volume as determined using 3-dimensional (3D) image-reconstruction software. We also investigated the association between the response to induction therapy on one hand and the complete resection and survival rates on the other. The effect of neoadjuvant therapy was also examined in patients with locally advanced thymic neoplasms, and their outcomes were compared to those of patients treated only by surgery.

## Materials and methods

**Fig 1:**
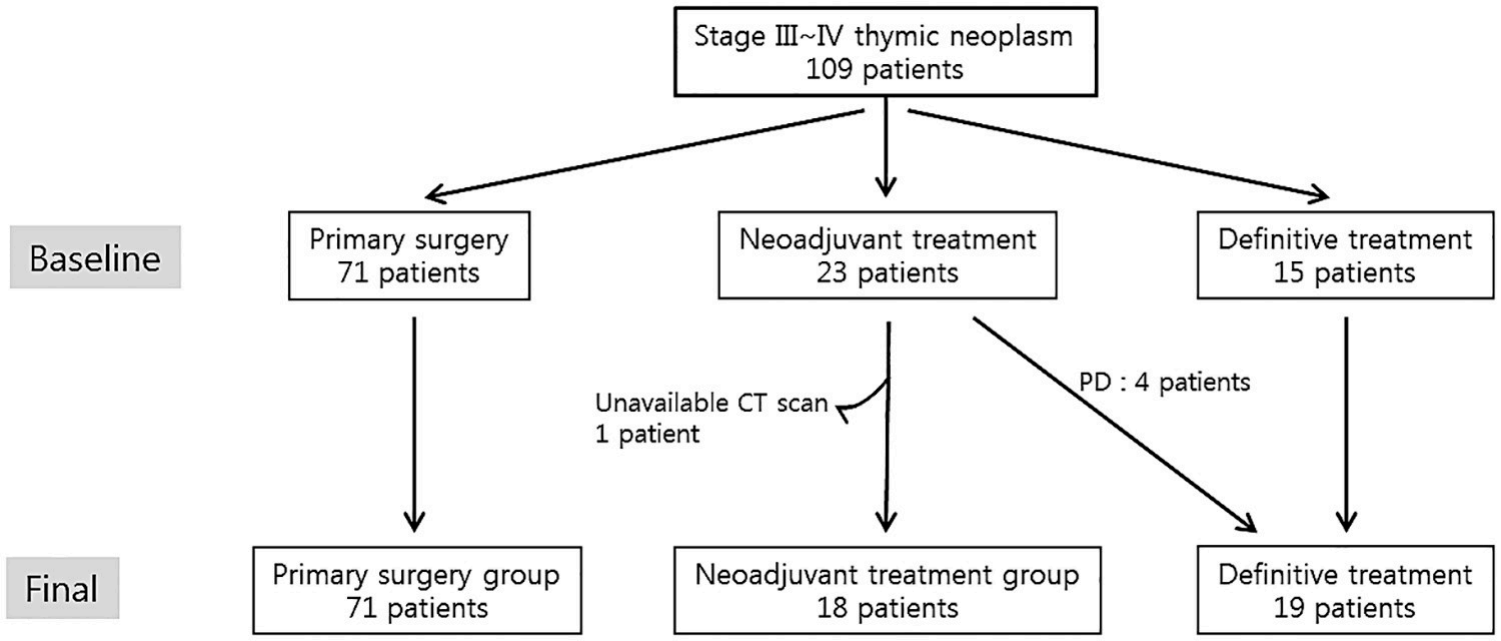
Flowchart of patient selection for the study.

Patients who underwent surgical resection for pathologic Masaoka-Koga stage III–IV thymic neoplasms at Yonsei University Severance Hospital between January 1, 2000 and December 31, 2013 were enrolled in this study. Patients with stage IVb disease who had apparent distant metastases were excluded, while those with pleural and/or intrathoracic lymph node metastasis were included. There were initially 109 patients with stage III or IV thymic neoplasms; their initial and final group distributions are shown in Fig 1. Four of the patients who underwent neoadjuvant treatment during the study period exhibited progressive disease, and their tumors were thereby deemed inoperable; therefore, they were reclassified into the definitive chemotherapy/chemoradiotherapy group, which ultimately comprised 19 patients. Furthermore, 1 patient undergoing neoadjuvant treatment was excluded because of unavailable preoperative CT data. The neoadjuvant group included patients with clinically unresectable disease, including tumors with extensive mediastinal involvement such as with the great vessels, chest wall, and lung as observed on CT.

A combination regimen of cisplatin, doxorubicin, vincristine, and cyclophosphamide (the ADOC regimen) was administered to 4 patients; cyclophosphamide, doxorubicin, and cisplatin (the CAP regimen) to 10 patients; and other forms of cisplatin-based chemotherapy to 4 patients. Most induction chemotherapy regimens before and after 2010 were ADOC and CAP, respectively; 5 patients received concurrent chemoradiotherapy.

**Fig 2:**
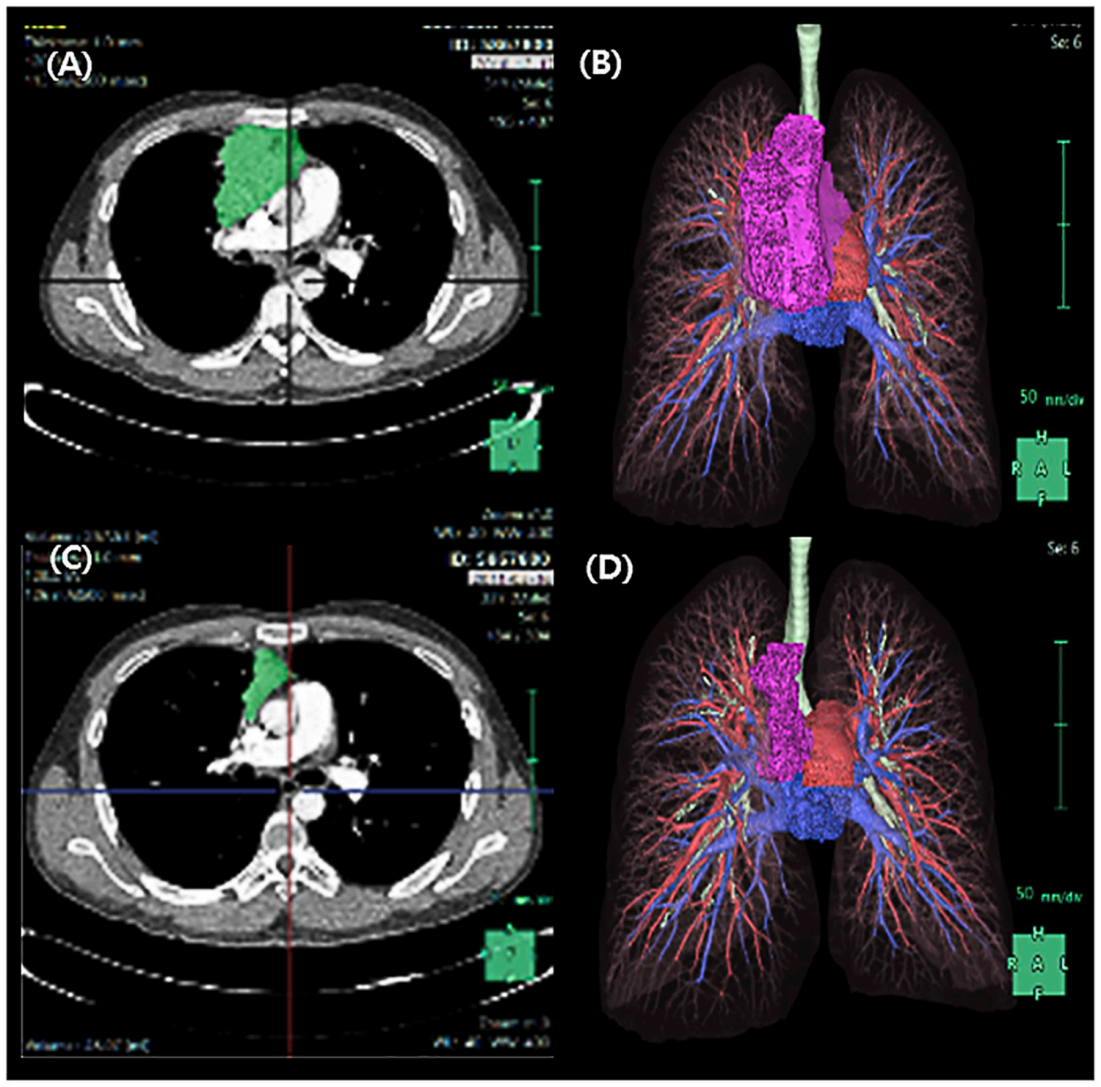
Measurement of thymic neoplasm size using the Synapse 3D software. A and B baseline, C and D: post-neoadjuvant therapy.

The response to induction therapy was evaluated by measuring the maximum tumor diameter on CT scans obtained within 4 weeks after the last chemotherapy cycle. Patients with progressive disease (PD), stable disease (SD), or complete remission based on CT findings underwent surgical resection after induction therapy. The response to induction therapy was defined according to the World Health Organization criteria: complete remission, complete disappearance of clinical and radiological evidence of disease; partial remission, ≥50% reduction in the sum of the diameters of all measured lesions; SD, regression of <50% of the mass, with no new lesions appearing; and PD, an increase of >25% in the sum of the lesions or the estimated size of non-measurable lesions, or the appearance of new lesions. Chest CT with or without contrast (1–5 mm thickness) was used to measure tumor sizes and to perform 3D reconstruction. Responsiveness was described based on the total volume of the mass (Fig 2) as measured using the Synapse 3D software (FujiFilm, Japan).

Surgery, which involved either sternotomy or minimally invasive surgery (video-assisted or robot-assisted thoracoscopic surgery with no rib or sternal spreading), was performed at a single center. Total thymectomy or thymomectomy was performed with a sufficient surgical margin in addition to en bloc removal of any of the involved adjacent mediastinal structures: the pericardium, pleura, lung, phrenic nerve, great vessel, and diaphragm.

Adjuvant therapy was administered to 83 patients (93.3%), including 15 in the neoadjuvant group, to control locoregional recurrence.

Follow-up and clinical outcome data were collected from the patients’ medical records. This retrospective study was approved by the Institutional Review Board of Severance Hospital (IRB 4-2018-0118).

Analytical data included baseline characteristics, postoperative outcomes, and survival. Baseline and post-neoadjuvant therapy tumor volumes in the neoadjuvant group were compared. Overall survival time was defined as the interval between the date of surgery and that of death from any cause. The disease-free survival time was defined as the interval between the date of surgery and that of recurrence or death from any cause. Statistical analyses were conducted using the SPSS software (ver. 23.0; IBM Corp., Armonk, NY, USA). Clinical and pathological parameters are reported as means ± standard deviation for continuous variables and as frequencies (%) for categorical variables. Student’s t-test was used to compare continuous variables, while the chi-square test was used to compare categorical variables. Comparisons between initial and post-induction therapy tumor volumes were determined using the Wilcoxon signed-rank test. Logistic regression was used to perform univariate analyses to identify the risk factors for incomplete resection, while Cox regression analysis was performed to identify the risk factors for disease recurrence in consideration of the time factor. Disease-free and overall survival rates were estimated using the Kaplan-Meier method. Statistical significance was defined as a p-value <0.05.

## Results

The baseline characteristics of the patients are shown in Table 1. The mean age was 53.89 ± 12.66 years in the primary surgery group and 48.28 ± 11.68 years in the neoadjuvant group (p = 0.092). The prevalence of myasthenia gravis, maximum length of the mass, and clinical stage differed between the 2 groups, but not significantly. The number of invaded mediastinal structures adjacent to the tumors was higher in the neoadjuvant group than in the primary surgery group (2.61 ± 1.42 vs. 1.27 ± 1.09, p<0.001); details of these mediastinal structures are shown in Table 1. Invasion of the phrenic nerve was reported in 11 patients (15.5%) in the primary surgery group and in 8 (44.4%) in the neoadjuvant group (p = 0.007). Invasion extents of the mediastinal pleura, innominate vein, and aorta differed significantly between the neoadjuvant and primary surgery groups.

**Table 1:** Baseline patient characteristics.

	Primary surgery (n = 71)	Neoadjuvant therapy (n = 18)	p-value
Sex			0.686
Male	47 (66.2%)	11 (61.1%)	
Female	24 (33.8%)	7 (38.9%)	
Age (years)	53.89±12.66	48.28±11.68	0.092
ECOG PS score			0.610
0	69 (98.6%)	18 (100%)	
1	1 (1.4%)	0	
Presence of MG	14 (19.7%)	1 (5.6%)	0.152
Maximal length on CT (cm)	6.64±2.85	7.31±1.70	0.222
Number of mediastinal invasions (observed with CT)	1.27±1.09	2.61±1.42	0.000*
Lung	30 (42.3%)	8 (44.4%)	0.867
Chest wall	1 (1.4%)	1 (5.6%)	0.658
Pericardium	36 (50.7%)	12 (66.7%)	0.225
Phrenic nerve	11 (15.5%)	8 (44.4%)	0.007*
Mediastinal pleura	1 (1.4%)	2 (11.1%)	0.042*
Innominate vein	6 (8.5%)	9 (50.0%)	0.000*
Superior vena cava	4 (5.6%)	3 (16.7%)	0.081
Aorta	0	2 (11.1%)	0.004*
Clinical Masaoka-Koga stage			0.703
I	5 (7.0%)	0	
III	52 (73.2%)	13 (72.2%)	
IVa	9 (12.7%)	3 (16.7%)	
IVb	5 (7.0%)	2 (11.1%)	

CT, computed tomography; ECOG PS, Eastern Cooperative Oncology Group performance status; MG, myasthenia gravis

Surgery consisted of sternotomy in most patients. Twelve patients (13.48%) underwent minimally invasive surgery; moreover, 63 patients in the primary surgery group (88.7%) and 15 in the neoadjuvant group (83.3%) underwent concurrent procedures (p = 0.534). Combined phrenic nerve and great vessel resections were performed more often in the neoadjuvant group than in the primary surgery group (phrenic nerves: 50% vs. 21.1%, p = 0.014; great vessel: 22.2% vs. 5.6%, p = 0.028). The resected great vessels included the aorta, innominate artery, and superior vena cava. Surgical durations and blood loss volumes were not significantly different between the 2 groups. Complete tumor resection was achieved in 51 (71.8%) and 12 (66.7%) of the patients in the primary surgery and neoadjuvant groups, respectively (p = 0.604) (Table 2). The reason for incomplete resection was a positive resection margin in 19 patients in the primary surgery group (26.8%) and in 5 patients in the neoadjuvant group (27.8%). Other reasons were dissemination in 1 patient in the primary surgery group (1.4%) and massive aortal invasion in 1 patient in the neoadjuvant group (5.6%). Perioperative outcomes including hospital stay, complications, mortality, and pathologic stage were not significantly different between the 2 groups (Table 2).

**Table 2:** Operative results and perioperative outcomes.

	Primary surgery (n = 71)	Neoadjuvant (n = 18)	p-value
MIS	10 (14.1%)	2 (11.1%)	0.741
Concurrent procedure	63 (88.7%)	15 (83.3%)	0.534
Lung	43 (60.6%)	12 (66.7%)	0.634
Pericardium	41 (57.7%)	11 (61.1%)	0.796
Diaphragm	1 (1.4%)	1 (5.6%)	0.289
Phrenic nerve	15 (21.1%)	9 (50.0%)	0.014*
Innominate vein	12 (16.9%)	5 (27.8%)	0.294
Great vessels	4 (5.6%)	4 (22.2%)	0.028*
Operation time (min)	168 (30–500)	227.5 (73–428)	0.053
Blood loss (mL)	200 (0–5300)	365 (0–3500)	0.474
Resection type			0.604
R0	51 (71.8%)	12 (66.7%)	
R1	19 (26.8%)	5 (27.8%)	
R2	1 (1.4%)	1 (5.6%)	
ICU admission	49 (71.0%)	10 (55.6%)	0.211
Complication	7 (10.0%)	1 (5.6%)	0.798
Operative mortality	0	0	
Hospital stay (median, days)	9.0 (3–55)	7.5(5–31)	0.338
Thymic carcinoma	33 (46.5%)	8 (44.4%)	0.545
Adjuvant therapy	68 (95.8%)	15 (83.3%)	0.094
Histologic type			0.405
WHO type A	1 (1.4%)	0	
WHO type AB	1 (1.4%)	0	
WHO type B1	3 (4.2%)	2 (11.1%)	
WHO type B2	6 (8.5%)	4 (22.2%)	
WHO type B3	27 (38%)	4 (22.2%)	
WHO type C	28 (39.4%)	8 (44.4%)	
Neuroendocrine	5 (7.0%)	0	
Pathologic M-K stage			0.849
III	49 (69.0%)	12 (66.7%)	
IVa	12 (16.9%)	4 (22.2%)	
IVb	10 (14.1%)	2 (11.1%)	
Recurrence	24 (33.8%)	10 (55.6%)	0.090

ICU, intensive care unit; MIS, minimally invasive surgery; M-K, Masaoka–Koga; WHO, World Health Organization.

We performed subgroup analysis in the neoadjuvant group to evaluate the therapy’s effectiveness. The mean tumor volumes in this group were 206.08 ± 132.32 cm^3^ at baseline and 81.25 ± 71.24 cm^3^ post-neoadjuvant therapy (p = 0.001); neoadjuvant therapy was associated with a significant reduction in tumor volume (delta volume: 124.82 ± 139.21 cm^3^, p = 0.001). However, the tumor diameters post-neoadjuvant therapy were not significantly different from their baseline values. Moreover, the extent of reductions in diameter lengths were not significantly different in patients who underwent complete vs. incomplete resections (Table 3). In terms of tumor volume, 13 patients (72.2%) achieved partial remission, 3 (16.7%) achieved SD, and 2 (11.1%) achieved PD. Thirteen patients (72.2%) achieved R0 resection. Initial tumor volumes were larger in the incomplete resection group than the complete resection group (311.23 cm^3^ vs. 153.50 cm^3^, p = 0.012); however, post-neoadjuvant therapy tumor volumes were not significantly different between the 2 groups (95.69 ± 36.65 cm^3^ vs. 74.04 ± 84.04 cm^3^, p = 0.458). Hence, large tumor volumes at baseline were more associated with incomplete resection than tumor volumes post-neoadjuvant therapy (Table 3).

**Table 3:** Subgroup analysis of patients in the neoadjuvant group.

	Complete resection (n = 13)	Incomplete resection (n = 5)	p-value
Baseline tumor volume (cm^3^)	153.50±56.37	311.23±180.65	0.012*
Post-neoadjuvant tumor volume (cm^3^)	74.04±84.04	95.69±36.65	0.458
Δ volume (cm^3^)	79.47±84.67	215.54±187.90	0.047*
Baseline tumor diameter (cm)	6.82±1.22	8.30±2.20	0.080
Post-neoadjuvant tumor diameter (cm)	5.08±2.51	7.33±2.99	0.111
Δ diameter (cm)	1.73±2.72	0.97±3.59	0.632

We performed univariate analysis of a number of factors to determine their association with complete resection. Undergoing neoadjuvant therapy was not associated with a higher rate of R0 resections (odds ratio [OR] 0.784, p = 0.667). Moreover, the number of invaded mediastinal structures and extensive resection of the adjacent structures were also not significantly associated with higher complete resection rates (OR 0.744, p = 0.105 and OR 2.262, p = 0.214, respectively) (Table 4).

**Table 4:** Univariate analysis of factors influencing R0 resection.

	R0 resection	
	OR (95% CI)	p-value
Age	0.971 (0.935–1.009)	0.130
Sex (male = 1)	0.504 (0.197–1.290)	0.153
Neoadjuvant treatment (none = 1)	0.784 (0.259–2.375)	0.667
Number of invaded mediastinal sites	0.744 (0.520–1.064)	0.105
Concurrent procedure during surgery (none = 1)	2.262 (0.624–8.200)	0.214
Pathologic M-K stage (stage III = 1)		
IVa	0.644 (0.203–2.047)	0.456
IVb	1.159 (0.280–4.802)	0.839

CI, confidence interval; M-K, Masaoka-Koga; OR, odds ratio

Thirty-four patients (38.2%) experienced recurrence (24 [33.8%] in the primary surgery group and 10 [55.6%] in the neoadjuvant group; p = 0.090) (Table 2). There were no significant differences in the sites of recurrence in the primary surgery vs. neoadjuvant groups (locoregional recurrence, 18 [75.0%] vs. 8 [80.0%]; distant recurrence, 6 [25.0%] vs. 2 [20.0%]; p = 0.754). On Cox-regression analysis, neoadjuvant therapy did not significantly influence the rate of recurrence (HR 2.519, p = 0.118) (Table 5).

**Table 5:** Cox regression analysis of factors associated with recurrence.

	Recurrence	
	HR (95% CI)	p-value
Age	0.961 (0.929–0.995)	0.025*
Sex (male = 1)	0.618 (0.277–1.382)	0.241
Neoadjuvant treatment (none = 1)	2.519 (0.792–8.009)	0.118
No. of invaded mediastinal sites	0.796 (0.532–1.192)	0.268
Concurrent procedure during surgery (none = 1)	3.944 (0.802–19.399)	0.091
R0 resection (not achieved = 1)	0.880 (0.353–2.197)	0.785
Adjuvant therapy (not performed = 1)	1.935 (0.502–7.454)	0.338
Pathologic M-K stage (stage III = 1)		
IVa	0.899 (0.366–2.208)	0.817
IVb	3.318 (1.148–9.591)	0.027*

CI, confidence interval; M-K, Masaoka-Koga; HR, Hazard ratio

**Fig 3:**
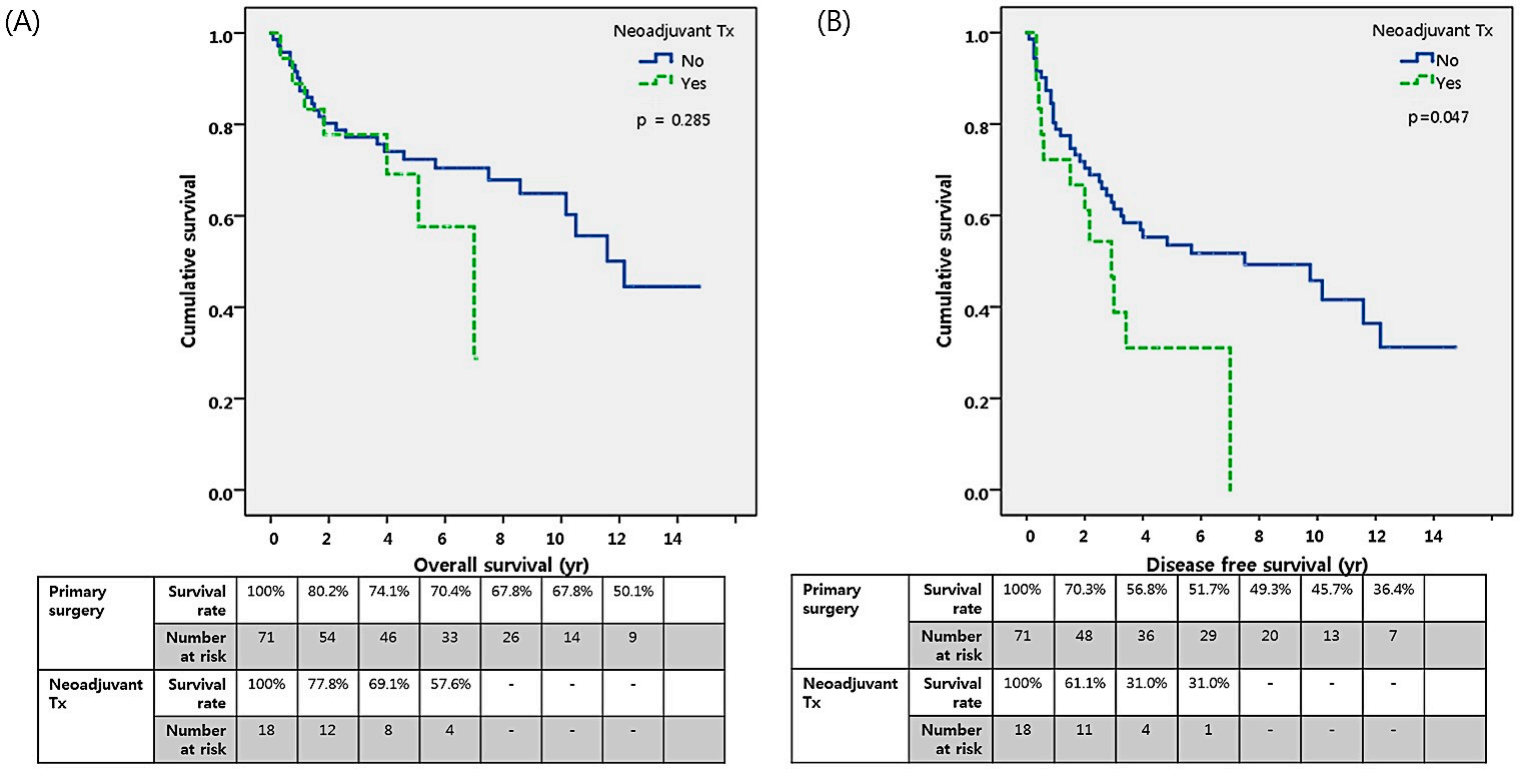
Kaplan-Meier plots of survival among patients in the primary surgery group vs. the neoadjuvant therapy group: (A) Overall survival (3-year, 77.2% vs. 77.8%; 5-year, 72.4% vs. 69.1%, p = 0.285); (B) Disease-free survival (3-year, 61.4% vs. 38.8%; 5-year, 53.5% vs. 31.0%; p = 0.047*).

The median follow-up time for the patients overall was 5.1 years (range, 0–14.8 years), and the 5-year survival rate of all patients was 71.7% (S1 Fig). The median follow-up time for the primary surgery group was 5.9 years (range, 0–14.8 years) while that for the neoadjuvant group was 3.9 years (range, 0.3–7.1 years). The 5-year disease-free survival rate was significantly higher in the primary surgery group than in the neoadjuvant therapy group (53.5% vs. 31.0%, p = 0.047), while the 5-year overall survival rate was not significantly different between these 2 groups (72.4% vs. 69.1%, p = 0.285) based on Kaplan-Meier analysis (Fig 3). On Cox regression analysis, none of the factors were found to significantly influence overall survival; pathologic stage was the only factor significantly associated with disease-free survival (hazard ratio 5.485, p<0.001).

## Discussion

To date, no consensus oncological treatment strategies have been developed for stage III–IV thymic tumors [1]. Although these cancers have been shown to be chemosensitive, conflicting data have been reported regarding the outcomes of patients with advanced disease who underwent induction therapy [8, 9]. While many studies have found that induction therapy confers a survival advantage, others have concluded that it does not [1].

In this study, we evaluated the effect of neoadjuvant therapy on thymic tumors as determined by their volumes. A 3D reconstruction program was used to calculate the volume of each tumor before and after treatment, and the results were then used to determine the response rate, which in turn was compared to the survival data. Neoadjuvant therapy conferred a significant reduction in tumor volume; however, the R0 resection rates did not differ significantly between patients in the primary surgery group and those in the neoadjuvant therapy group. Nevertheless, mediastinal invasion was more frequent, and the surgeries more complex, in the latter group. Subgroup analysis of the neoadjuvant group revealed that a small baseline tumor volume was significantly associated with achieving complete resection, while the post-neoadjuvant therapy tumor volume was not. Moreover, neoadjuvant treatment was found to confer no significant advantage in terms of overall survival or disease recurrence rate.

Induction therapy is generally well tolerated and has an acceptable toxicity profile [5]; it is administered to reduce both the tumor size and the extent of its infiltration, thus allowing for easier excision during surgery and fewer complications [10]. In this study, neoadjuvant treatment did not produce significant changes in intraoperative bleeding volumes or surgical durations compared to primary surgery (365 vs. 200 mL, p = 0.474; and 227.5 vs. 168 minutes. vs, p = 0.053; respectively). There were also no significant differences in terms of either the risk of surgical complications (10.0% vs. 5.6%, p = 0.798) or mortality (none in either group). However, given the large baseline tumor volumes in patients in the neoadjuvant therapy group, the achievement of operative outcomes comparable to those of primary surgery indicates that neoadjuvant therapy could offer the advantage of facilitating surgery by reducing both the tumor volume and extent of invasion.

In patients with Masaoka-Koga stage III–IV disease, a preoperative workup does not predict the likelihood of surgical complete resection. Furthermore, high-resolution contrast-enhanced CT is useful for demarcating the anatomic relationships between the tumors and adjacent organs, but does not detect microscopic invasion or adhesion [5, 10], thus complicating the determination of the tumor’s clinical disease stage and resectability. If incomplete resection is expected in large-volume tumors that have likely invaded adjacent organs, surgical resection may be improved by first reducing the extent of the tumor with preoperative treatment.

This study had several limitations. First, it was a retrospective non-randomized study with a small sample size and an unequal number of patients in the 2 groups. Second, we did not perform pathologic evaluation of the effect of neoadjuvant treatment. Nevertheless, our results showed that neoadjuvant treatment dramatically reduces tumor volumes in patients with advanced thymic neoplasms, in whom complete surgical resection is likely to be difficult. Thus, neoadjuvant treatment facilitated the surgical procedure, thereby attaining R0 resection, improving survival, and achieving outcomes that are comparable to those of patients undergoing primary surgery.

## Supporting information

S1 Fig Overall survival in total patients. (TIF)
