# Supplementary figures and images for: Neoadjuvant therapy for thymic neoplasms reduces tumor volume per 3D-reconstructed images but does not improve the complete resection rate
Source: PLoS One. 2019 Mar 26;14(3):e0214291. doi: 10.1371/journal.pone.0214291 (PMC6435136; doi:10.1371/journal.pone.0214291)

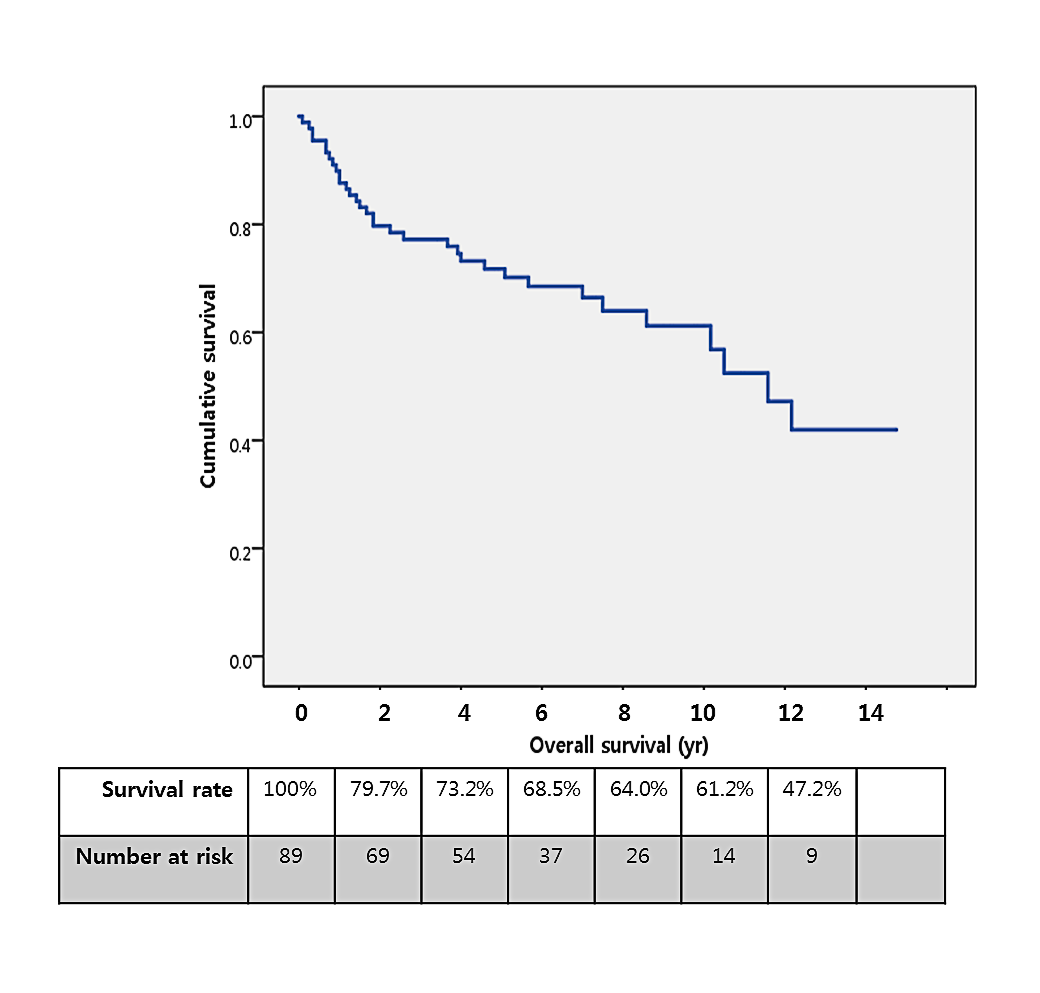

Supplement: S1 Fig — (TIF) [file pone.0214291.s001.tif]
